# Molecular tools prove little auks from Svalbard are extremely selective for *Calanus glacialis* even when exposed to Atlantification

**DOI:** 10.1038/s41598-023-40131-7

**Published:** 2023-08-22

**Authors:** Kaja Balazy, Emilia Trudnowska, Katarzyna Wojczulanis-Jakubas, Dariusz Jakubas, Kim Præbel, Marvin Choquet, Melissa M. Brandner, Mads Schultz, Julie Bitz-Thorsen, Rafał Boehnke, Marlena Szeligowska, Sébastien Descamps, Hallvard Strøm, Katarzyna Błachowiak-Samołyk

**Affiliations:** 1 Department of Marine Ecology, Institute of Oceanology, Polish Academy of Sciences, Powstancow Warszawy 55, 81-222 Sopot, Poland; 2 Department of Vertebrate Ecology and Zoology, Faculty of Biology, University of Gdansk, 80-309 Gdansk, Poland; 3 Norwegian College of Fishery Science, UiT The Arctic University of Norway, Tromsø, Norway; 4 Faculty of Biosciences and Aquaculture, Nord University, 8049 Bodø, Norway; 5 Department of Medical Biochemistry and Microbiology, Uppsala University, Uppsala, Sweden; 6 Norwegian Polar Institute (NPI), Fram Centre, 9296 Tromsø, Norway

**Keywords:** Ecological genetics, Climate-change ecology, Marine biology

## Abstract

Two *Calanus* species, *C*. *glacialis* and *C*. *finmarchicus*, due to different life strategies and environmental preferences act as an ecological indicators of Arctic Atlantification. Their high lipid content makes them important food source for higher trophic levels of Arctic ecosystems including the most abundant Northern Hemisphere's seabird, the little auk (*Alle alle*). Recent studies indicate a critical need for the use of molecular methods to reliably identify these two sympatric *Calanus* species. We performed genetic and morphology-based identification of 2600 *Calanus* individuals collected in little auks foraging grounds and diet in summer seasons 2019–2021 in regions of Svalbard with varying levels of Atlantification. Genetic identification proved that 40% of *Calanus* individuals were wrongly classified as *C*. *finmarchicus* according to morphology-based identification in both types of samples. The diet of little auks consisted almost entirely of *C*. *glacialis* even in more Atlantified regions. Due to the substantial bias in morphology-based identification, we expect that the scale of the northern expansion of boreal *C*. *finmarchicus* may have been largely overestimated and that higher costs for birds exposed to Atlantification could be mostly driven by a decrease in the size of *C*. *glacialis* rather than by shift from *C*. *glacialis* to *C*. *finmarchicus*.

## Introduction

Arctic regions are warming faster than other regions in the world^1–3^. Anomalous Atlantic Water inflow into the Arctic Ocean causes a progressive transition of Arctic waters to a state more closely resembling that of the Atlantic (so called ‘Atlantification’)^4,5^. New environmental conditions pose a challenge to the species living there, being an opportunity or a threat, depending on their ecological flexibility. So far advantage of species with flexible adaptive traits with progressing warming has been observed^6^. To better understand the Atlantification process it is crucial to track responses of some species with strong environmental preferences, which are often used as ecological indicators of Arctic Atlantification. These are either typically Atlantic species, which presence indicate an increased inflow of warmer waters to the Arctic, such as *Oithona atlantica*, or typically Arctic species, such as larger copepods, adapted to cold environments, being especially sensitive to the changes of the environment in which they live^7^.

In the region of Svalbard, acting as a gateway to the Arctic Ocean, there are two endemic Arctic species, which serve as ‘model species’ for studying the response of High Arctic organisms to ongoing changes: copepods of the genus *Calanus*^8^ and the zooplanktivorous seabird, the little auk (or dovekie) *Alle alle* feeding on them^9^. *Calanus* species are especially important components of the Arctic food web, due to their extremely high lipid content^10^. The fifth copepodite stage (CV) of cold water copepods (*Calanus hyperboreus and C. glacialis*) is characterized by high energy content because of huge amount of fat collected before overwintering^11^. Thus, this type of prey constitutes a perfect food source for the little auk^12–14^, dominating its diet even in 80–90% both in terms of numbers and biomass^15^. Little auks are particularly important to Arctic ecosystems because of their role as ecological engineers. Their very high numbers of breeding pairs collect food for their chicks at sea and deposit large amounts of guano in terrestrial ecosystem. This behaviour makes them the main fertilizers of ornithogenic tundra^16,17^. As a highly selective predator, they are predicted to be particularly vulnerable to climate change due to the loss of the majority of suitable foraging grounds in the future^18,19^.

These assumptions are based on the predicted replacement of typically Arctic species, such as *C*. *glacialis*, by boreal counterparts, such as *C*. *finmarchicus.* And indeed, the numerical importance of copepods associated with warmer waters has been observed to increase along with progressive warming^20–22^. As higher lipid content was attributed to larger *C*. *glacialis* compared to smaller, less energetically profitable boreal *C*. *finmarchicus*^11^, these changes were considered to be unfavourable to little auks preferring cold water *C. glacialis*. A different view was proposed by Renaud et al.^23^ suggesting that lipid content in *Calanus* is not species-specific, but body size-specific. This size-based trait approach resulted from demonstrating the previous misidentification of two *Calanus* species. Traditionally *C*. *glacialis* and *C*. *finmarchicus* were distinguished by their morphological characteristics linking particular copepodite stage with the proper range of prosome length^13,24^. Studies conducted over the last decade using molecular tools have shown that the separation of these two species exclusively on the basis of morphologic criteria without genetic identification is unreliable, due to considerable inter-species prosome length overlap^23,25–28^. Both *Calanus* species, although morphologically very similar, have different life history strategies and distribution ranges, consequently they also differ in response to ongoing climate change, so their proper identification is crucial in order to understand the large-scale changes which are currently taking place in the Arctic environment^29^.

Several studies have confirmed the misidentification of *Calanus* species in net samples collected at sea in the single stations of the North Atlantic and Arctic Oceans including Svalbard fjords^29^. However, these studies have been focused neither on the little auk foraging grounds nor the little auk diet composition. Very high variation of *Calanus* sizes in various locations has been demonstrated, including the presence of large *C*. *finmarchicus* individuals, misclassified as *C*. *glacialis*, in the warmer Svalbard fjords^25,28^ or reduced body size of *C*. *glacialis*^25,30^. However, it is still unknown how this variability affects the diet of little auks, considered their selective targeting larger zooplankters^31^. This is especially important as the composition of their diet act as one of the indicators of climate change in the High-Arctic environment^9^. Therefore, in light of the confirmed wide misidentification of both *Calanus* species, the significant need emerged to determine their actual contribution in the diet of the little auks, especially that all models predicting the future of these bird populations in the Arctic^18,32^ are based solely on the standard morphological classification of *Calanus* species. This study fills gaps in knowledge about key species from Svalbard region, by (1) re-evaluating the use of *C*. *finmarchicus* and *C*. *glacialis* body (prosome) length to assess the influence of the Atlantic Ocean in the Arctic Ocean, (2) determining factors influencing *Calanus* body length, and (3) comparing proportions of *Calanus* species in net and diet content to estimate variability in prey availability and preferences.

## Material and methods

### The study area

**Figure 1:**
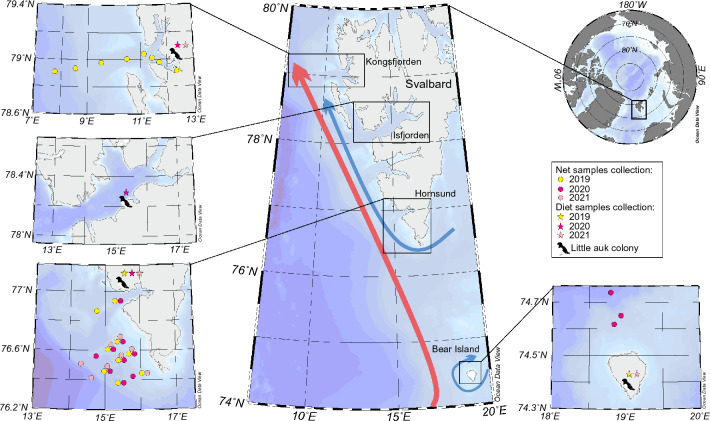
Location of the net sampling stations in Kongsfjorden, Hornsund and Bjørnøya (dots) and little auk colonies in Kongsfjorden, Isfjorden, Hornsund and Bjørnøya (stars) marked with different colours for the three years of research 2019–2021 in Svalbard, Norway. The arrows show the dominating ocean currents: warm West Spitsbergen Current (red arrow), cold Spitsbergen Polar Current (SPC) (blue arrow). Background: bathymetry. Map was prepared with Ocean Data View software v. 5.2.1 (http://odv.awi.de)^38^.

The study was conducted in four regions of Svalbard that host large little auks breeding colonies. These are three fjords on the west coast of Spitsbergen—Hornsund, Isfjorden and Kongsfjorden and on the southernmost island of the Svalbard archipelago—Bear Island (Bjørnøya) (Fig. 1). The study locations are characterized by hydrographic regimes in the foraging areas and different exposure to Atlantification. The Hornsund area is situated on the south-western tip of Spitsbergen and is influenced mainly by the cold and fresh coastal current named the Spitsbergen Polar Current (SPC). Despite the increased influx of Atlantic waters observed in this area over the last two decades, the strong controlling effect of Arctic Water, transported by the SPC helps Hornsund preserves its Arctic character^33^. The high-caloric zooplankton prevailing in the cold water masses serves as attractive food for the zooplanktivores. That is why the breeding aggregations of little auks in Hornsund is considered one of the largest in Svalbard^34^. Contrary to Hornsund, little auk colonies in Isfjorden and Kongsfjorden are much smaller^34^. Kongsfjorden located in the north is exposed to advection from the warmer Atlantic water of the West Spitsbergen Current (WSC) and receives twice as much Atlantic water than Hornsund^35^. In turn, hydrological conditions inside Isfjorden, the largest fjord on west Spitsbergen, are in a state of dynamic equilibrium between contrasting large water masses, but in the era of climate change, they are increasingly subjected to "Atlantification"^36^. Bear Island (Bjørnøya), with the southernmost Svalbard population of little auk, is surrounded by cold Arctic Water which from the south and west mix with a branch of the warm North Atlantic Current. The island's close proximity to the Polar Front, rich in biological production, makes it an attractive nesting and foraging area for the seabirds including little auks^37^.

### Zooplankton sampling

Net samples were collected during three summer seasons 2019–2021 in three locations (Bjørnøya, Hornsund and Kongsfjorden) located in traditional foraging areas of little auks. The latter areas of the little auks from these colonies are known from previous studies based on at-sea surveys and/or GPS-tracking^18,39^. Exact sampling dates, the number of samples collected and the locations of the sampling stations are provided on Fig. 1 and Table 1. At each zooplankton sampling station at each study area samples were collected using a WP2-type net (0.25 m^2^ opening area) fitted with filtering gauze of 180 µm mesh size. All zooplankton samples were collected from the mid-surface water layer (i.e., upper 50 m), corresponding to the little auk maximal diving depth (38–50 m)^40,41^. After collection, samples were split into two subsamples. One subsample was preserved in 4% formaldehyde solution in borax-buffered seawater for a traditional, morphometrics-based taxonomic identification. The second subsample was preserved in 70–96% ethanol for genetic identification. Ethanol was changed after the first 24 h. All samples were then transported to the laboratory for further analysis.

**Table 1:** Sampling dates for net samples in three regions (*HOR* Hornsund, *BJR* Bjørnøya, *KGF* Kongsfjorden) with a number of samples (*N* samp.) and a number of genetically identified individuals (*N* ind.) with two different genetic identification methods; mitochondrial cytochrome c oxidase gene (COI) and Insertion or Deletion motifs (InDels).

Net samples						
Year	Site	Date	*N* samp.	*N* ind.	*Gen.* ID COI	*Gen.* ID InDels
2019	HOR	30 Jul–1 Aug	9	279	154	125
	KGF	8–11 Aug	8	242	151	91
2020	HOR	13–14 Jul	10	311	311	–
	BJR	12 Jul	3	89	89	–
2021	HOR	28–29 Jul	8	182	–	182
Total			37	1103	705	398

Diet samples were collected during three summer seasons 2019–2021 in four breeding colonies (Bjørnøya, Hornsund, Isfjorden and Kongsfjorden). Exact sampling dates, the number of samples collected and the locations of the sampling stations are provided on Fig. 1 and Table 2. Food loads were collected from adult birds returning from the foraging areas to the colony, from a gular pouch (a sublingual sack below the tongue), where adults transport food for the chick^42^. Food delivered to chicks and stored in the gular pouch is fresh enabling prey identification and measurements. Birds were captured in the colonies using noose-carpet, loops or mist-nets. Food was gently removed from the gular pouches with a small metal spoon. Same as with the net samples, zooplankton from food loads collected from little auks were split into two subsamples and preserved as described above. We performed all the fieldwork by permission of the Norwegian Animal Research Committee (19/32026) and the Governor of Svalbard (20/00373-2). All the birds were handled with the utmost care and released without any harm after the sampling. All procedures on little auks were carried out in accordance with guidelines for the use of animals in research^43^. Reporting in the manuscript follows the recommendations of the ARRIVE (Animal Research: Research: Reporting of In Vivo Experiments) guidelines^44^.

**Table 2:** Sampling dates for diet samples in four regions (*HOR* Hornsund, *BJR* Bjørnøya, *ISF* Isfjorden, *KGF* Kongsfjorden) with a number of samples (*N* samp.) and a number of genetically identified individuals (*N* ind.) with Insertion or Deletion motifs method (*Gen*. ID InDels).

Diet samples					
Year	Site	Date	*N* samp.	*N* ind.	*Gen. ID*
2019	HOR	29–30 Jul	15	475	InDels
	BJR	27–31 Jul	8	255	InDels
2020	HOR	21 Jul	5	160	InDels
	KGF	17 Jul	2	66	InDels
	ISF	10 Jul	3	94	InDels
2021	HOR	28–29 Jul	11	348	InDels
	KGF	27–29 Jul	3	71	InDels
	BJR	27 Jul	1	34	InDels
Total			48	1503	

### Sea temperature measurements

To characterize sea temperature (hereafter ST) in little auks foraging areas the continuous oscillatory profiles, from the surface to 50 m depth, were performed with a conductivity–temperature–depth sensor (CTD; SBE 911plus, Seabird Electronics Inc., Washington, DC, USA). CTD data were collected under the long-term monitoring program AREX. For the stations near Bjørnøya, where no CTD measurements were taken, daily sea surface temperature (SST) data from Sentinel-1 from the Norwegian Meteorological Institute were used (https://cryo.met.no).

On the basis of ST and SST data at-sea stations from all locations have been classified into three water domains: Cold Water (CW) < 4 °C, Transitional Water (TRW) 4–6 °C and Warm Water (WW) > 6 °C. Cold water masses temperature corresponds to surface water temperature on the Arctic side of the front, and warm water masses temperature corresponds to surface water temperature on the Atlantic side of the front during Arctic summer^45^. The classification of water masses also refers to the temperature limit of *C*. *glacialis*, where < 4 °C are considered optimal for this species, while ~ 6 °C is considered as its upper thermal tolerance^46,47^.

### Laboratory analysis

All samples preserved in a 4% formaldehyde solution were subject to standard taxonomic analysis following the instructions given by Kwasniewski et al.^13^. First, zooplankters larger than 0.5 cm were identified, counted and removed from the sample. Then, 2 mL subsamples were taken from each sample according to the sub-sampling method^48^. Subsamples were taken until at least 400 individuals were counted. All organisms were identified to the lowest possible taxonomic level, typically species. *Calanus* copepodite stages, which differ in the number of urosome segments and pairs of legs were identified according to criteria described by Kwasniewski et al.^24^.

All samples preserved in ethanol were screened in order to select individuals for molecular species identification. Sub-samples were taken from each sample until 32 randomly chosen individuals of *Calanus* fifth copepodite stage (CV) were counted. Each selected *Calanus* CV individual was measured from the tip of the cephalosome to the distal lateral end of the last thoracic somite (prosome length and hereafter body length) using the ruler in the eye-piece of a stereomicroscope to measure directly (resolution 1 unit). It was then classified as *C*. *glacialis* or *C*. *finmarchicus* according to the size range for the species specified in Kwasniewki et al.^24^. Then each individual was placed in ethanol for further molecular species identification. *Calanus hyperboreus* as a deep-water, oceanic species^49–51^ is very rarely caught in planktonic nets dedicated for sampling mesozooplankton fraction^52^. It is mainly associated with Greenland waters constituing substantial component of chick diets in the eastern^32,53,54^ and northwest Greenland^55,56^. In food loads of little auks inhabiting Svalbard colonies is found frequently, but in small numbers acting as supplementary diet component^57,58^. Therefore in our study *Calanus* group studied consists only of *C*. *glacialis* or *C*. *finmarchicus*, while *C*. *hyperboreus* has been classified as supplementary diet component.

### Molecular species identification

Once all *Calanus* individuals were measured and rinsed in ultra-pure water, molecular identification was carried out. Individuals from all little auk diet samples and from 15 samples from at sea-hauls (1901 individuals) (Tables 1, 2) were identified to species using a set of six nuclear molecular markers of the type InDel (Insertion or Deletion motifs)^59^, following the simplified protocol described in Choquet et al.^29^. Briefly, DNA was extracted from the two excised antennules of each individual using a HotSHOT protocol^60–62^ and used for the PCR amplification of the six InDels. Resulting amplicons were sized using a 3500xL Genetic Analyzer (Applied Biosystems), generating a species-specific profile. Individuals from the remaining samples from at-sea hauls (705 individuals) (Table 1) were analysed for species identification using DNA sequencing of a fragment of the subunit one of the mitochondrial cytochrome c oxidase gene (COI). This method has been used and validated in the studies of Coguiec et al.^63^, where *Calanus* species including *C*. *glacialis* and *C*. *finmarchicus* were distinguished. First, DNA was extracted using HotSHOT alkaline lysis method, that recently have been shown to be an ideal method for extracting zooplankton because it minimizes DNA loss in all steps of the extraction^64^. Each individual was placed in 20 μL Alkaline Lysis Reagent and heated at 95 °C for 30 min to lyse the individuals and their cells. The suspension was then pH-neutralized by adding 20 μL of neutralization buffer and the DNA extracts were kept at 4 °C until amplification. A ~ 313 base pair fragment of the mitochondrial cytochrome C oxidase I (COI) gene was amplified by polymerase chain reaction (PCR) using the highly degenerated Leray-XT primer-set^65^. The amplification was conducted in a single 10 μL PCR using 1 μL DNA template, 5 μL AmpliTaq Gold Master mix, 0.08 μL Bovine Serum Albumin (20 μg/μL), 0.5 μL of each of the individually-tagged Leray-XT forward and reverse primers (5 μM), and 2.92 μL H_2_O. Single-step PCR followed an initial denaturing step at 95 °C for 10 min, 35 cycles of 94 °C for 1 min, 45 °C for 1 min and 72 °C for 1 min, and a final extension step of 72 °C for 5 min. PCR products with twin sample tags attached at both ends were pooled and purified using MinElute columns (Qiagen). The Illumina library was prepared from the DNA pool using the NextFlex PCR-free library preparation kit (Perkin-Elmer), which incorporates Illumina sequence adapters by ligation. The resulting library was sequenced in an Illumina MiSeq 300 PE. The Leray-XT library was sequenced with 1% PhiX on the Illumina MiSeq platform in the UiT The Arctic University of Norway, using V3 chemistry (2 × 250-bp paired-ends). The DNA sequencing reads from the barcoded sea-haul individuals were processed following the Mjolnir pipeline (https://github.com/uit-metabarcoding/MJOLNIR), with the same parameters and filtering as in Turon et al.^66^, resulting in a molecular operational taxonomic units (MOTU) table with taxonomic identities. The MOTU table was then filtered to eliminate prokaryotic (or unassignable), and non-marine sequences (Supplementary Table S1). The identity for the *Calanus* spp. in each barcode sample was assigned based on a threshold of > 90% of the reads, belonging to the assigned species. The samples for which the species ID from COI was unclear were further confirmed with InDels.

### Data analysis

Accuracy of the morphological identification was calculated as the percent between the number of correct species assessments in relation to the number of all species assessments using genetic identification. The accuracy was calculated separately for each species in different sites, water domains and little auks’ colonies.

A distance-based linear model (DistLM)^67^ was used to analyse the relationships between the body length of *Calanus* individuals genetically identified to species (response variable) and sea temperature (explanatory variable).

Multivariate nonparametric permutational ANOVA (PERMANOVA)^67^ was used to test differences in the body length of *Calanus* species in different water domains and the percentage of *C*. *glacialis* vs. *C*. *finmarchicus* in little auks diet between colonies. These analyses were based only on individuals identified morphologically and genetically to the species. To show the size distribution of *Calanus* species identified genetically the 1d kernel density (R function geom_density) estimates of the dominating size were calculated in R software version 4.1.3^68^.

Relation between ST and proportion of *C*. *glacialis* vs. *C*. *finmarchicus* identified genetically were illustrated using DIVA interpolation in Ocean Data View software^38^.

In order to better understand the selectivity of little auks from individual colonies and to link the composition of its diet with particular water masses, apart from *Calanus* individuals, supplementary diet items were analysed. PERMANOVA test was used to test variation in the community of non-*Calanus* items in the diet samples of little auks from different colonies. Canonical analysis of principal coordinates (CAP) was used to visualize the variability of zooplankton composition in little auks diet samples along the two axes that best discriminated among the groups of samples defined by the colony. Pearson correlation vectors of groups of species’ relative abundances with axes were overlaid on the CAP plot. Non-Calanus species were categorized into four groups: Arctic, Boreo-Arctic, Boreal and Ubiquitous according to biogeographic origin according to classification provided in Weydmann et al.^20^. Prior to the analyses, data was normalized by square-root-transformation^69^. The calculation of the Pseudo-F and P values were based on 999 permutations of the residuals under a reduced model^70^. PERMANOVA, DISTLM and CAP were performed in Primer v7 and Permanova software^67,71^.

### Ethical statement

We performed all the fieldwork by permission of the Norwegian Animal Research Committee (19/32026) and the Governor of Svalbard (20/00373-2). All the birds were handled with the utmost care and released without any harm after the sampling. All procedures on Little Auks were carried out in accordance with guidelines for the use of animals in research^43^. Reporting in the manuscript follows the recommendations of the ARRIVE (Animal Research: Research: Reporting of In Vivo Experiments) guidelines^44^.

## Results

### Accuracy of *Calanus copepodite* stage CV species identification

**Figure 2:**
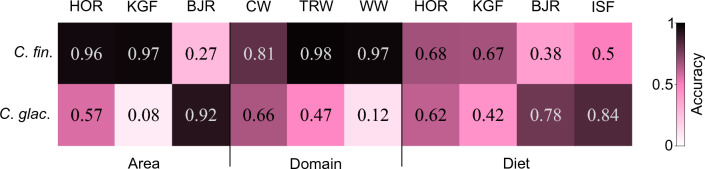
Accuracy of morphological species identification in different sites (*HOR* Hornsund, *KGF* Kongsfjorden, *BJR* Bjørnøya), water domains (*CW* Cold Water, *TRW* Transitional Water, *WW* Warm Water) and little auks’ colonies (*HOR* Hornsund, *KGF* Kongsfjorden, *BJR* Bjørnøya, *ISF* Isfjorden) confirmed by genetics.

In zooplankton samples from at-sea hauls in the little auks foraging areas accuracy calculated for each sampling area separately showed that the species identification according to size-based morphological procedures was almost fully accurate for *C*. *finmarchicus* in Hornsund and Kongsfjorden and for *C*. *glacialis* in Bjørnøya (Fig. 2). Much lower accuracy was obtained for *C*. *glacialis* in Hornsund (57%) and, Kongsfjorden (8%), where a large portion of the individuals had reduced body sizes. Relatively low accuracy (27%) was also obtained for *C*. *finmarchicus* in Bjørnøya, where most of the misidentified *C*. *finmarchicus* individuals turned out to be larger than the size limit of *C*. *finmarchicus*. Accuracy calculated for water domains for *C*. *finmarchicus* individuals was high in all domains (81–98%), in contrast to the relatively high misidentification obtained for *C*. *glacialis,* especially in Atlantic water domain (Fig. 2).

For diet samples collected from adult little auks overall accuracy for morphological identification of *Calanus* species confirmed by genetics was 65% for *C*. *glacialis* and 63% for *C*. *finmarchicus*. The highest contribution of misidentified *C*. *glacialis* was recorded in Kongsfjorden (58%) and the lowest in Isfjorden (16%). In Kongsfjorden and Isfjorden only one individual from those identified as *C*. *glacialis* turned out to be a large *C*. *finmarchicus*. In Bjørnøya, among overall 222 individuals identified by morphometrics as *C*. *glacialis* only 10 individuals (4.5%) turned out to be large *C*. *finmarchicus*, while in Hornsund 22 large individuals from 588 studied (3.7%) were wrongly classified as *C*. *glacialis* by morphometrics.

### Temperature effect on *Calanus copepodite* stage CV body size and species distribution

**Figure 3:**
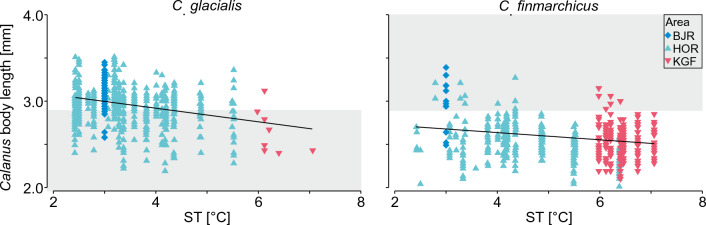
The relations between seawater temperature (ST) and body (prosome) length of *C*. *glacialis* and *C*. *finmarchicus* copepodite stage CV. Markers represent individuals measured at foraging grounds (*HOR* Hornsund, *KGF* Kongsfjorden, *BJR* Bjørnøya). The grey background represents species delimitation (i.e. individuals not classified as a given species) according to a standard morphometrics-based classification^24^. Black line represents linear model.

There was a significant effect of seawater temperature on the body length of the two *Calanus* species investigated (Fig. 3). The DistLM procedure confirmed that ST explained 32% of the total variability observed in the body length of *C*. *glacialis* and *C*. *finmarchicus* combined (DISTLM, Pseudo-F = 502.96, P = 0.001, R^2^ = 0.32). When the two species were considered separately, for *C*. *glacialis* ST explained 8% of the total variability observed in the body length (Pseudo-F = 46.88, P = 0.001, R^2^ = 0.08), while for *C*. *finmarchicus* 5% (Pseudo-F = 28.38, P = 0.001, R^2^ = 0.05). The largest and most numerous individuals of *C*. *glacialis* were recorded at ST < 4.5 °C (Fig. 3). On Bjørnøya, *C*. *glacialis* body length ranged from 2.6 to 3.4 mm, while its population consisted almost entirely of individuals larger than 2.9 mm. The largest *C*. *glacialis* individuals were observed in Hornsund (3.5 mm), but a considerable proportion of its entire population in this area was smaller than 2.9 mm, with the smallest individuals with 2.2 mm body length. In Kongsfjorden where the ST was above 6 °C, *C*. *glacialis* reached body length from 2.3 to 3.2 mm, while almost all *C*. *glacialis* individuals attained small body sizes < 2.9 mm. *C. finmarchicus* individuals were generally small and most numerous at temperatures higher than 6 °C SST, especially in warm Kongsfjorden. Only a few individuals of *C. finmarchicus* larger than 2.9 mm were observed in all areas, with the largest in Bjørnøya, (Fig. 3).

**Figure 4:**
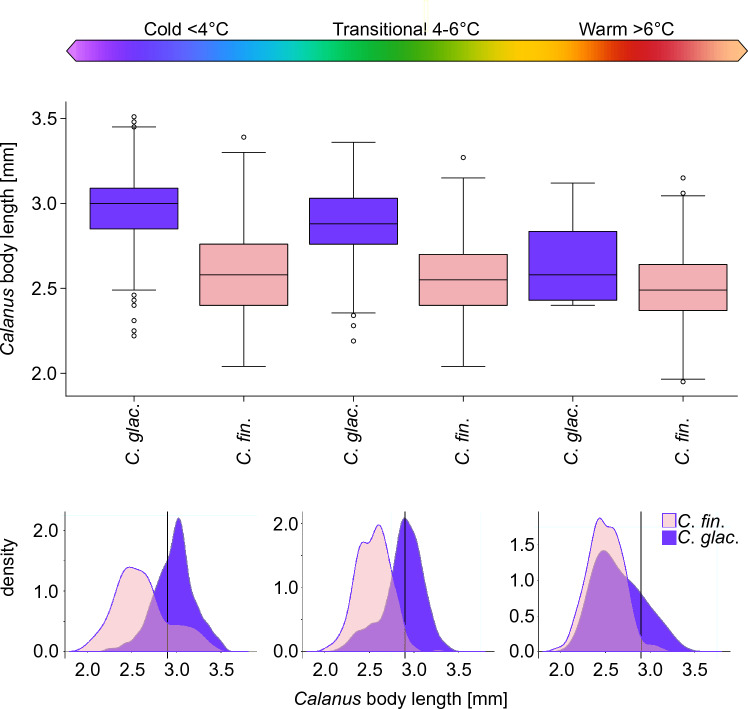
Body (prosome) length of *Calanus glacialis* (“*C*. *glac.*”) and *Calanus finmarchicus* (“*C*. *fin*.”) copepodite stage CV in different water domains; Cold, Transitional and Warm (upper panel). Black lines in the boxes show the median, box represents percentiles, whiskers indicate ranges, dots represent values outside the range. Size distribution of *C*. *glacialis* (purple) and *C*. *finmarchicus* (pink) in the corresponding water domains (lower panel). The solid black vertical lines represent species delimitation according to standard morphological classification^24^.

The body length distribution of *Calanus copepodite stages CV* differed between the two species in three water domains (PERMANOVA, MS = 0.8, Pseudo-F = 183.78, P = 0.001). These differences varied from the most pronounced in the Cold domain to similar sizes in the Warm domain (Fig. 4). Statistically significant differences confirmed by post-hoc test for PERMANOVA were not found only for *C*. *finmarchicus* in the Cold domain vs. *C*. *glacialis* in the Warm domain (P = 0.776), and *C*. *finmarchicus* in Transitional domain vs. *C*. *glacialis* in the Warm domain (P = 0.152). Comparing the differences in body size within individual *Calanus* species, both species were the largest in the Cold domain and the smallest in the Warm domain, while these differences were more pronounced for *C*. *glacialis* than for *C*. *finmarchicus*.

**Figure 5:**
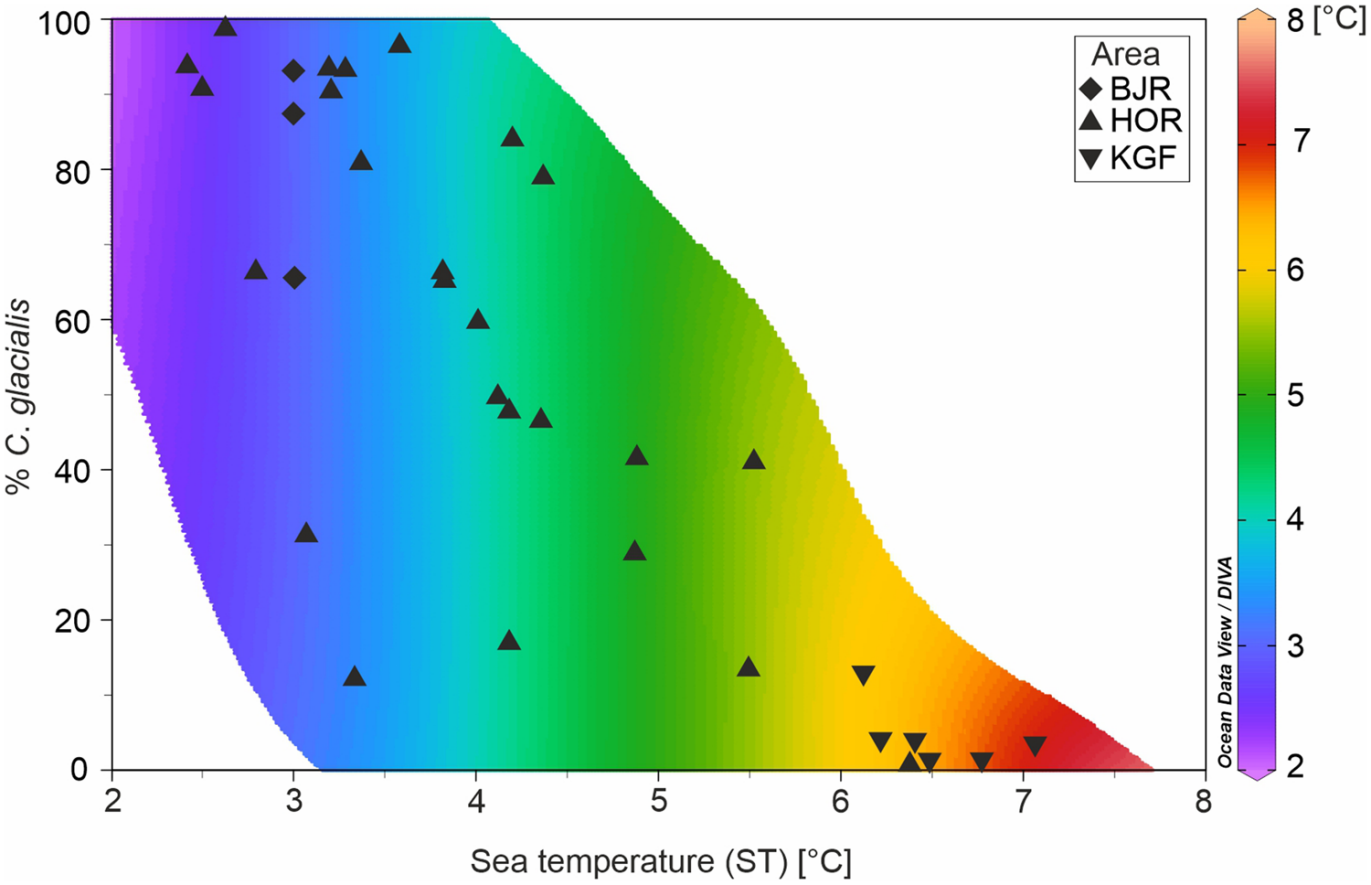
Contribution of *C*. *glacialis* vs. *C*. *finmarchicus* copepodite stage CV expressed by % *C*. *glacialis* in relation to Sea Temperature (ST) from upper 50 m water layer at stations (*HOR* Hornsund, *KGF* Kongsfjorden, *BJR* Bjørnøya) during studied summer seasons (2019–2021).

The relative roles of both *Calanus* species changes with ST measured at each station in Kongsfjorden, Hornsund and Bjørnøya area (Fig. 5). Kongsfjorden, where ST at each station was > 6 °C was clearly dominated by *C*. *finmarchicus* (87–100%). In Hornsund, a clear dominance of *C*. *glacialis* in the water < 4 °C ST was observed and its percentage share was higher with lower temperature. The balance of the share of both species occurred at a temperature of about 4–4.5 °C. A clear dominance of *C*. *finmarchicus* was observed at stations with a temperature > 5 °C. In Bjørnøya, all stations with SST ~ 3 °C were dominated by *C*. *glacialis* (65–93%).

### *Calanus* in little auks diet vs. foraging grounds

**Figure 6:**
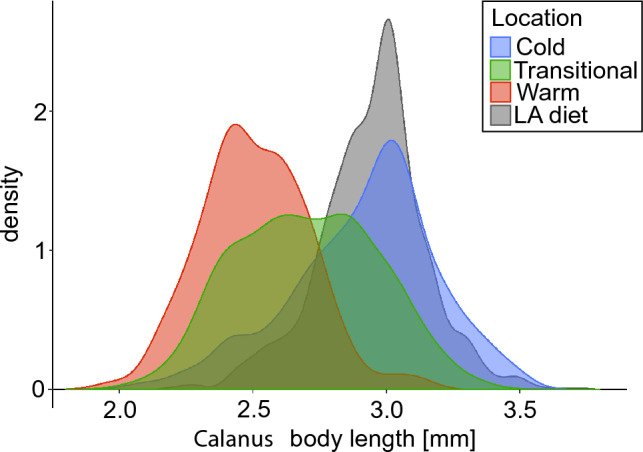
Size distribution of *Calanus* fifth copepodite stage (CV) (*C*. *glacialis* and *C*. *finmarchicus*) in different water domains (Cold, Transitional, Warm) and little auks diet samples (LA diet) from all studied stations and colonies in Svalbard. *Calanus* body length is the length of the prosome.

There is a clear match in the body length of *Calanus* in the diet of little auks with that observed in the Arctic domain, where the size distribution peaks of about 3.1 mm overlap completely (Fig. 6). Partial overlap also occurs between the sizes of *Calanus* in the diet and the Transitional domain, while the body length of copepods in Atlantic waters clearly differs from those observed in the diet of birds.

**Figure 7:**
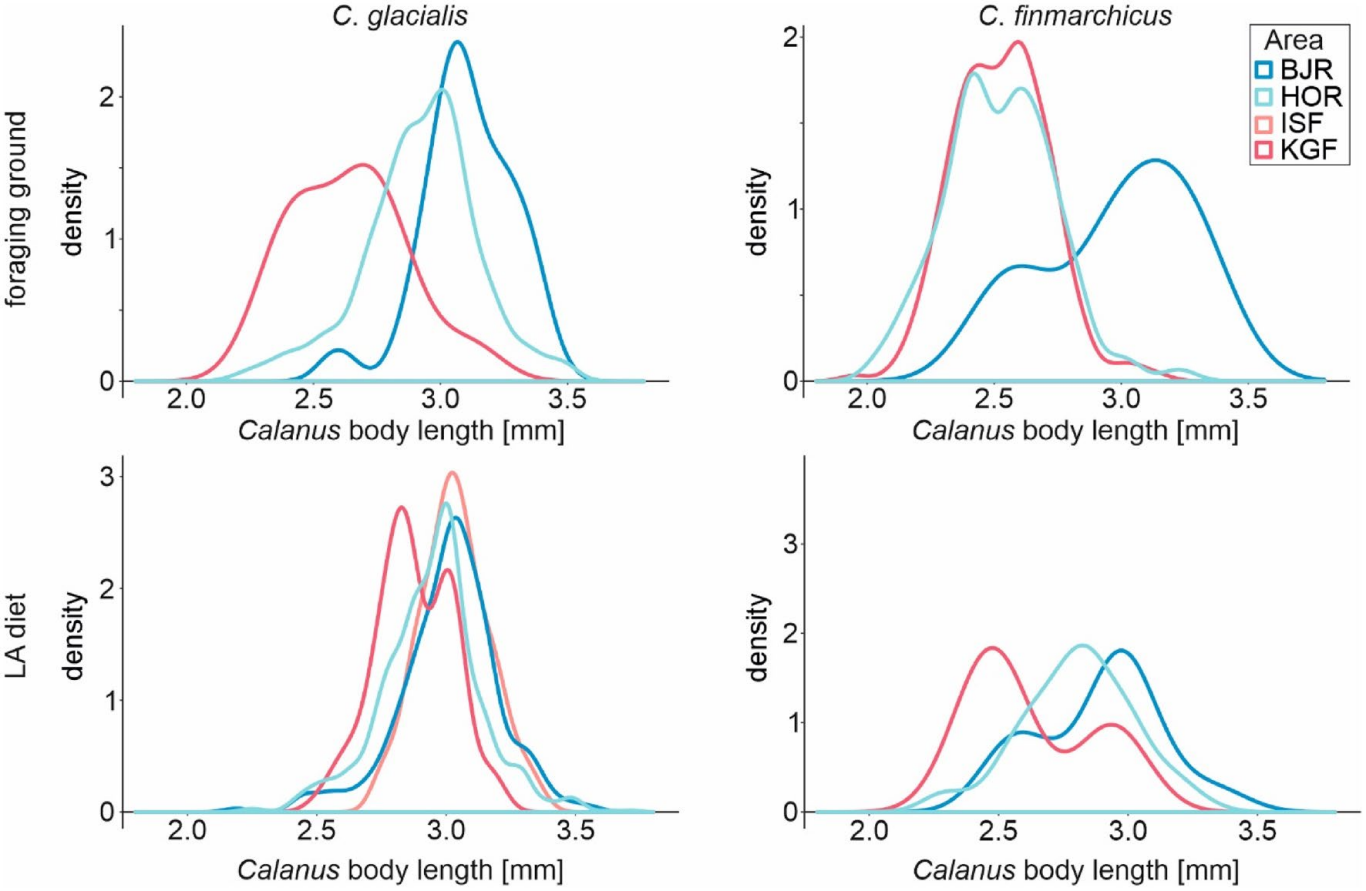
Size distribution of *C*. *glacialis* (left panel) and *C*. *finmarchicus* (right panel) copepodite stage CV at foraging grounds (upper panel) and little auks diet samples (lower panel) from all studied areas (*HOR* Hornsund, *KGF* Kongsfjorden, *BJR* Bjørnøya, *ISF* Isfjorden). The data from Isfjorden are shown only for *C*. *glacialis* in the little auks diet. *Calanus* body length is the length of the prosome. For *C*. *finmarchicus*, they were not included due to the small number of individuals (n = 2).

The diet of little auks from all colonies consisted of generally large individuals of both *Calanus* species (Fig. 7). Compared to the size distribution for *C*. *glacialis* in the foraging grounds, the same body length peak of approx. 3.0–3.1 mm of this species was observed in the diet of birds from all colonies expect Kongsfjorden where *C*. *glacialis* were smaller in the foraging ground than in the diet. A unique pattern different from other colonies was observed for the diet of birds from Kongsfjorden with two body size peaks of *Calanus*. Little auks in this colony picked for the largest but rare prey items (*C*. *glacialis* and *C*. *finmarchicus*) and more common but smaller ones. Generally little auks from all colonies chose larger individuals of *C*. *finmarchicus* (2.9 mm) than those that predominated in the foraging grounds (2.4–2.6 mm). When looking at the size distribution of both *Calanus* species in individual foraging grounds, the largest prey items were generally observed in the colder foraging areas of birds from Bjørnøya and Hornsund while the smallest were observed in the warmest Konsgfjorden (Fig. 7). PERMANOVA procedure confirmed the difference in the body length of both *Calanus* species in the diet of little auks between studied colonies (PERMANOVA, MS 45.06, Pseudo-F = 16.72, P = 0.001). The largest *C*. *glacialis* individuals with an average body length of ~ 3 mm were observed in the diet of birds from Bjørnøya and Isfjorden, slightly smaller from Hornsund and the smallest ones from Kongsfjorden colony. For *C*. *finmarchicus* individuals which were generally smaller than *C*. *glacialis*, the same body size pattern in prey items was observed in the studied colonies.

**Figure 8:**
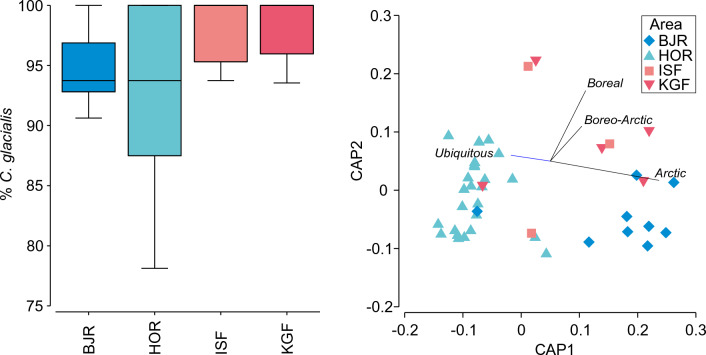
Box plot presenting proportion of *Calanus glacialis* copepodite stage CV in *Calanus* group (*C*. *glacialis* and *C*. *finmarchicus*) (left panel). Black lines in the boxes show the median, boxes represent percentiles, whiskers indicate ranges, dots represent values outside the range. CAP ordinations (right panel) showing the groups of samples providing the best discrimination, defined by little auks colony and based on Bray–Curtis similarities of supplementary diet items relative abundances classified into four groups of species origin (Arctic, Boreo-Arctic, Boreal, Ubiquitous). Symbols represent little auk colonies from different locations (*HOR* Hornsund, *KGF* Kongsfjorden, *BJR* Bjørnøya). Vectors indicate the Pearson correlation of the group best correlated with the ordination coordinates. Vector length corresponds to the correlation value.

Regardless of the colony, *C*. *glacialis* clearly dominated the diet over *C*. *finmarchicus*, constituting on average over 94% of all identified *Calanus* individuals (Fig. 8). The percentage of *C*. *glacialis* vs. *C*. *finmarchicus* in diet samples did not differ between colonies (PERMANOVA, MS 17.60, Pseudo-F = 1.62, P = 0.175). However, the diet of birds from Isfjorden and Kongsfjorden were more homogeneous in species selected compared to Hornsund which had a much wider range of values with even < 80% of *C*. *glacialis* in some diet samples.

### Supplementary diet items

Examination of the variation in the community of supplementary diet items indicated a marked separation among the studied colonies (PERMANOVA, MS 6744.8, Pseudo-F = 5.00, P = 0.001) (Fig. 8). The greatest differences were observed between the diet samples from Hornsund and Bjørnøya (PERMANOVA pair-wise tests, P = 0.001), compared to very similar samples from Kongsfjorden and Isfjorden (PERMANOVA pair-wise tests, P = 1), which is clearly illustrated by the colony-oriented CAP ordination (Fig. 8). The benthic larvae taxa, especially *Eupagurus* and *Hyas* larvae had the greatest impact on the diversity of samples from Hornsund, while typical Arctic species like *Calanus hyperboreus*, *Apherusa glacialis* or *Themisto libellula* were the most numerous in supplementary diet components of birds from Bjørnøya. The diet of the birds from Konsgfjorden and Isfjorden colonies were more heterogenous and mainly supplemented with boreo-Arctic *T*. *abyssorum* or typically Atlantic species like *Thysanoessa longicaudata* or *Pandalus borealis* zoea, which were not noted in the diet of birds from other colonies. However, some samples were similar in composition to those of Hornsund and Bjørnøya containing typically Arctic diet items, like *T*. *libellula* or *C*. *hyperboreus*.

## Discussion

This study confirms that molecular methods are necessary to ground truth the range of *C*. *finmarchicus* increasing role in the Svalbard waters and its assumed increased contribution to little auks diet. Thanks to proper, genetically proven species identification it was possible to present the level of *Calanus* size variability across regions, different water domains as wells as between available food resources at sea in comparison to the prey selection of little auks in their diet. Molecular methods are becoming an increasingly popular tool supporting traditional methods of species identification. They are especially helpful when morphological identification of fragmented or partially digested organisms are very difficult or even impossible^72–74^. In the case of two sibling *Calanus* species, they are well-preserved in both net samples and little auks diet samples thanks to the unique method of storing food by the birds in a special sublingual pouch, which enables precise body measurements of undigested individuals. Thus, their identification based mainly on differences in their body (prosome) length has not caused any problems until recently. Reports of misidentification generated a sudden need to study the scale of this phenomenon with greater resolution to answer the question of how this relates to our knowledge of *Calanus*-feeding higher trophic levels, especially little auks considered to be indicators of climate change in the Arctic.

Research to date has shown that the correct identification of two sympatric *Calanus* species cannot be based solely on morphological characteristics, because its effectiveness varies between regions and environmental conditions^25^. Particularly pronounced discrepancy was demonstrated in fjords along the Norwegian coast where the size range of both species overlapped completely^25^. In Svalbard waters, the effectiveness of the morphometrics-based distinction between *C*. *glacialis* and *C*. *finmarchicus* was considered high with 97% and 100% accuracy at the Arctic and Frontal stations in Hornsund fjord, respectively^30^. However, a potential problem of underestimating *C*. *glacialis* has been demonstrated due to reported cases of its reduced body length in different water masses^25,30^. Our study clearly indicates that the misidentification problem is more serious than previously thought. As many as 40% of individuals genetically designated as *C*. *glacialis* from little auks foraging grounds located in Svalbard waters were smaller than expected based on morphological criteria and could thus be wrongly identified as *C. finmarchicus*. On the other hand, only 5% of *C*. *finmarchicus* individuals reached a larger body size than predicted by the traditional body size-based classification. Therefore, the scale of the underestimation of *C*. *glacialis* demonstrated in the present study may have serious implications for the interpretation of ongoing processes in marine High Arctic ecosystems. Most studies to date have linked the process of progressive Atlantification with the replacement of the arctic *C*. *glacialis* by the boreal *C*. *finmarchicus* as a result of increased biomass of *C*. *finmarchicus* in the Arctic Ocean observed in long-term plankton data^20–22^. All these datasets, however, are based on traditional morphological classification, when molecular methods were not used to identify *Calanus* species. We can therefore assume that these studies to some extent may have overestimated the pace and scale of the northern expansion of *C*. *finmarchicus* with progressive warming. Presumably, some portion of copepods identified as *C*. *finmarchicus* in fact represented smaller individuals of *C*. *glacialis*. Our studies may thus rather have documented a change in size of *C*. *glacialis* through time rather than an increase in *C*. *finmarchicus* abundance and opens the question, whether *C*. *glacialis* has always been smaller or it is its size response to higher temperatures? *Calanus* spp. body size was found to be inversely related to temperature^75^. It is predicted that *Calanus* body size reduction will systematically proceed with further climate warming^23,76^. Interestingly, in our study, this relationship was more pronounced for *C*. *glacialis*, which, together with its wide size variability, indicates greater size plasticity of this species. This would also be indicated by the results of recent studies on the metabolic activity of enzymes in both species which indicates the advantage of *C*. *glacialis* over *C*. *finmarchicus* with more efficient lipid anabolism, which may be of particular benefit in High-Arctic unpredictable environments^77^. Moreover, the authors of that study questioned whether *C*. *finmarchicus* could actually establish itself in the Arctic seas and replace *C*. *glacialis*.

We found a predominance of *C*. *glacialis* in cold waters < 4.5 °C near Hornsund and Bjørnøya, while warmer waters of Atlantic origin west of Hornsund and entirely in Kongsfjorden were completely dominated by *C*. *finmarchicus*. This is in line with the recognized maximum tolerance temperature of 6 °C for *C*. *glacialis* and better adaptation of *C*. *finmarchicus* to life at higher temperatures^47,78,79^. However, one must bear in mind that in the present study we only included the most important for little auks, energy rich—the fifth copepodite stage (CV) of *Calanus*, and the remaining stages are necessary to fully examine the population status in the near future. Cold waters surrounding Hornsund and Bjørnøya rich in *C*. *glacialis* are potentially valuable foraging grounds for the little auks^72,80^, in contrast to the intensively Atlantified area of Kongsfjorden, where *C*. *glacialis* is extremely scarce.

**Figure 9:**
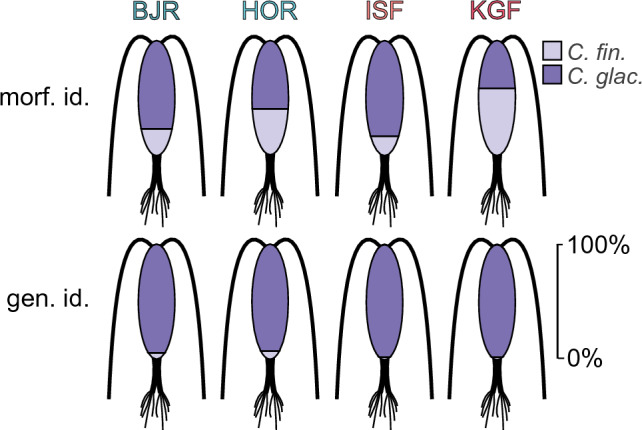
Comparison of the percentage of *C*. *finmarchicus* and *C*. *glacialis* copepodite stage CV in the diet of little auks from different colonies according to morphological (top) and genetic identification (bottom) of *Calanus* species.

Until now, little auks were considered to feed almost exclusively on large, energy-rich *Calanus* copepods, mainly *C*. *glacialis* in the Spitsbergen region and *C*. *hyperboreus* in Greenland^81^. *Calanus finmarchicus* as smaller and less energetically valuable was considered as a diet supplement^57^*.* Due to its Atlantic origin, it was considered to be an indicator of progressing Atlantification of feeding grounds^13^ and its higher numbers proved a lower quality of the birds' diet^19^. Therefore, *C*. *finmarchicus* constituted greater biomass in the diet of birds foraging in the areas with more Atlantic conditions^58^. About 35% of *C*. *glacialis* individuals in the diet of birds from all studied areas had reduced body sizes and could be wrongly identified as *C. finmarchicus* based only on morphometric criterion. Considering the highest number of small prey items in food samples from Kongsfjorden, one may expect the highest contribution of *C*. *finmarchicus* in the diet in this Atlantified region. However, genetic identification revealed different pattern—regardless of the location of the colony, little auks seem to rely almost entirely on *C*. *glacialis* which accounts for nearly 100% of their diet (Fig. 9). This is especially interesting when looking at the diet of birds from Kongsfjorden, where little auks experience the overwhelming dominance of *C*. *finmarchicus* in the foraging grounds. Despite selecting smaller, but common prey items they also picked for the largest but very rare ones, which was a unique pattern of foraging, different from other colonies.

This confirms the high prey selectivity of the little auks actively searching for larger *Calanus* individuals^14,31,82^. Our study suggests that such selectivity seems to be enhanced when the feeding grounds are strongly influenced by warm Atlantic water masses. Contrary to our expectations, the highest contribution of *C*. *finmarchicus* in some food loads was observed in birds from the ‘most Arctic’ Hornsund. At the same time, the abundance of *Calanus* spp. prey items in the diet of birds from this colony was the largest among the studied sites. This suggests that birds experiencing high prey availability in cost-effective distance from the colony are somewhat more tolerant to the addition of smaller, energetically suboptimal *C*. *finmarchicus*. It should also be mentioned that the misidentification of *C*. *finmarchicus* CV in the diet samples in the present study was also high (37%). Importantly, however, large *C*. *finmarchicus* in the little auk diet were not numerous (2%) in the studied years in all colonies. It strengthens the evidence for the aforementioned high prey selectivity of little auks for large copepods, as large *C*. *finmarchicus* individuals are in a clear minority compared to the smaller ones at the foraging grounds. All this leads to the conclusion that birds are unable to compensate for the scarcity of larger *Calanus* individuals by foraging higher abundances of smaller ones. This lack of adaptability confirms the concern that the transformation of trophic webs towards smaller zooplankton expected to be beneficial for other top predators, seems to be particularly unfavourable for little auks^23^, at least during the breeding period with the highest energy requirements. Moreover, given the presence of typically Arctic or open water species (*C*. *hyperboreus*, *T*. *libellula*) in the diet of birds from Konsgfjorden and Isfjorden, it can be assumed that these birds foraged in more distant foraging grounds^12^. There is a high cost associated with locomotion and foraging^83,84^, we assume that the search for valuable food by birds in areas exposed to Atlantification in the face of poor food quality near the colony, is associated with larger energy investments. Our study suggests that such higher costs could be actually mostly driven by a decrease in the size of *C*. *glacialis* rather than by a shift in species from *C*. *glacialis* to *C*. *finmarchicus*. This may negatively affect little auks which are considered flexible, but vulnerable to changing environmental conditions^18,19,85^. Our research shows that molecular methods are necessary not only to study the ecology of *Calanus* copepods per se, but also to gain significant knowledge in the broader context of studying the ecology of predators that prey on them, changing our view on the dynamics and direction of changes taking place in the increasingly Atlantified Arctic environment.

## Conclusions

Our study highlights the significant problem of underestimation of *C*. *glacialis* in the research on the ecology of *Calanus* species to date. We have shown that the pace and scale of the northern expansion of *C*. *finmarchicus* along with the progressive Atlantification may have been overestimated. The misidentification of *Calanus* species has also been indicated to have a significant influence on the interpretation of zooplanktivorous little auk plasticity in response to a warming environment. Genetic identification of *Calanus* individuals among little auks prey items revealed a stronger prey selectivity than reported before, regardless of the location of their colony. We also expect that higher costs for birds exposed to Atlantification could be actually mostly driven by a decrease in the size of *C*. *glacialis* rather than by a species shift from *C*. *glacialis* to *C*. *finmarchicus*. The results of our research show a strong need to incorporate molecular methods into future research on these important ecological indicators of Arctic Atlantification and re-evaluate the existing ones.

### Supplementary Information

Supplementary Information.

## Data Availability

The datasets analysed during the current study are available from the corresponding author on request.
